# Modelling nonlinear moderation effects with local structural equation modelling (LSEM): A non‐technical introduction

**DOI:** 10.1002/ijop.13259

**Published:** 2024-10-19

**Authors:** Tuo Liu, Ruyi Ding, Zhonghuang Su, Zixuan Peng, Andrea Hildebrandt

**Affiliations:** ^1^ Institute of Psychology Goethe‐Universität Frankfurt am Main Frankfurt Germany; ^2^ Department of Psychology Sun Yat‐Sen University Guangzhou China; ^3^ Department of Psychology Carl von Ossietzky Universität Oldenburg Oldenburg Germany

**Keywords:** Local structural equation modelling, Moderation, Nonlinearity

## Abstract

Understanding the differential strength of effects in the presence of a third variable, known as a moderation effect, is a common research goal in many psychological and behavioural science fields. If structural equation modelling is applied to test effects of interest, the investigation of differential strength of effects will typically ask how parameters of a latent variable model are influenced by categorical or continuous moderators, such as age, socio‐economic status, personality traits, etc. Traditional approaches to continuous moderators in SEMs predominantly address linear moderation effects, risking the oversight of nonlinear effects. Moreover, some approaches have methodological limitations, for example, the need to categorise moderators or to pre‐specify parametric forms of moderation. This tutorial introduces local structural equation modelling (LSEM) in a non‐technical way. LSEM is a nonparametric approach that allows the analysis of nonlinear moderation effects without the above‐mentioned limitations. Using an empirical dataset, we demonstrate the implementation of LSEM through the R‐sirt package, emphasising its versatility in both exploratory analysis of nonlinear moderation without prior knowledge and confirmatory testing of hypothesised moderation functions. The tutorial also addresses common modelling issues and extends the discussion to different application scenarios, demonstrating its flexibility.

Structural equation modelling (SEM) has proven to be a versatile tool in psychological science, particularly in elucidating the relationships between latent variables and their observable indicators (Bollen et al., 2022). In diverse subfields of psychology and behavioural sciences that commonly use SEMs, much attention has been paid to how the relationships between latent variables are moderated by a third variable, such as age, socio‐economic status, ability and personality traits (e.g., openness, neuroticism). Moderators, which alter the patterns of relationships between latent variables, can be either categorical (e.g., nationality) or continuous (such as age in months or years, socio‐economic status, personality traits, etc.) variables. SEM approaches for analysing the moderation effects of categorical variables, such as multigroup comparison, are familiar to most researchers. However, there is a need for researchers to become more proficient in analysing the effects of continuous moderators.

With this tutorial, we aim to enrich the methodological toolbox of applied psychological scientists by introducing local structural equation modelling (LSEM; Hildebrandt et al., 2009, 2016; Robitzsch, 2023), a useful method to address the moderation effect of a continuous variable. LSEM has the potential to reveal more intricate and detailed effects than the artificial categorisation of moderator, a practice that is suboptimal in previous SEM research, namely by categorising naturally continuous variables for the purpose of analysis, such as artificial dividing the ages in several bins. Furthermore, in much of the research that uses SEM to examine moderating effects, the implicit assumption is that these moderating effects are linear (Li, 2018). One reason for this assumption is possibly that in the seminal work of Baron and Kenny (1986), moderation has statistically been represented as a linear interaction between the independent variable and the moderator (Kraemer et al., 2008). While this linear assumption simplifies statistical modelling and facilitates a more accessible approach to data analysis (Penna et al., 2018), research on moderation effects rarely emphasises the necessity of testing whether the linearity assumption of moderation holds (Wu & Zumbo, 2008). Undoubtedly, this neglect in research practice raises concerns about misspecification and erroneous conclusions.

Given the versatility of SEM in examining the relationships between latent constructs and the theoretical importance of moderation analysis, this tutorial introduces LSEM as a flexible statistical modelling approach for analysing nonlinear moderation effects within the SEM framework. With this tutorial, we aim to enrich the methodological toolbox of applied psychological scientists. Specifically, as an approach without parametric assumptions like the linearity of a moderation, LSEM is particularly suitable for investigating nonlinear moderation effects with latent variables. LSEM is readily accessible to applied researchers through a user‐friendly function in the open‐source statistical software R (Robitzsch, 2023).

## EARLY APPROACHES TO ESTIMATING MODERATION IN SEM

Several early approaches have been explored to test the moderation effects of a continuous variable within the SEM frameworks. One prevalent approach is multiple‐group structural equation modelling (MG‐SEM; Evermann, 2010). In this approach, the SEM is fitted simultaneously to covariance matrices characteristic of different subsets of the data, each subset representing a specific value of the moderator and forming distinct groups (Gana & Broc, 2019). The inferential comparison of SEM parameters across these groups indicates moderation effects. If the number of groups was large and the grouping variable was at least on an ordinal scale, one can describe parameter differences across the groups with a linear or nonlinear function. This approach, however, originally proposed for categorical (nominal or ordinal) moderators with a rather small number of groups, faces challenges when applied to a continuous moderator with many potential values, which is more common in differential and developmental psychology (Burchinal et al., 2014; Gorrese & Ruggieri, 2012). The challenge is that the overall sample size in many applications may not be large enough to have sufficient observations at each potential value of a continuous moderator to estimate an MG‐SEM across as many groups as there are moderator values. To overcome this limitation, a typical research practice is to categorise continuous moderators artificially (Dimitruk et al., 2007), such as dividing age into young and old sub‐groups using a median split to enable the use of MG‐SEM. However, categorising continuous moderators is generally discouraged in the methodological literature (Cohen, 1983; MacCallum et al., 2002; Maxwell & Delaney, 1993). The disadvantages of such categorisation are threefold. First, it leads to a significant loss of information about individual differences within moderator groups. For example, when age is categorised into broader groups, such as childhood versus adolescence, specific differences within each group, such as 6 versus 7 years old in childhood or 12 versus 13 years old in adolescence, are no longer detectable. Second, the arbitrariness of categorisation cutoffs can profoundly influence the results (Hildebrandt et al., 2009). For instance, different researchers may set different thresholds for the same moderator, such as defining the age period of adolescence (Sawyer et al., 2018), and leading to inconsistent results across different studies. Third, and most importantly, in the context of nonlinear moderation in SEM, this practice might overlook nuanced nonlinear moderation patterns by only analysing too few groups (Hildebrandt et al., 2016). In order to thoroughly explore more nuanced moderation patterns, a fine segmentation of the moderator is necessary. Using age as an example again, a researcher would need to categorise age into small intervals and create many narrowly defined age groups. However, SEM requires large sample sizes to achieve adequate statistical power, especially as the number of estimated parameters increases (Cooper et al., 2019). Although there are more developments of small sample size (*n* < 100) solutions for SEM (Rosseel, 2020), *n* = 200 is often considered the minimum sample size needed for SEM (Boomsma, 1985). Thus, as mentioned above, a very large sample size (each age group *n* > 200) would be needed to ensure that an MG‐SEM has sufficient observations to reach adequate statistical power. The very large sample size poses serious feasibility challenges in many applied research areas, especially in resource‐intensive laboratory studies.

Another approach is moderated nonlinear factor analysis (MNLFA), which avoids categorising a continuous moderator by modelling SEM parameters as a function of this continuous moderator (Bauer, 2017; Bauer & Hussong, 2009, p. 20). Unlike MG‐SEM, MNLFA includes a moderator as a predictor of any SEM parameters (intercept, factor loading, variance, or path), allowing for nonlinear effects by adding higher‐order polynomial terms of a moderator (Tucker‐Drob, 2009). However, this method is limited by its reliance on a predefined parametric form of moderation. Researchers must specify the functional form of the moderation, such as linear, quadratic, or cubic (Jacoby, 2000), which is only advantageous when the form is known or predicted by a strong theory, which is often not the case. The challenge is, however, that the correct functional form of a moderation effect is almost always unknown, at least in new and innovative research fields, leading to the risk of misrepresenting the relationship in the data (Olaru et al., 2019). Additionally, the inherent polynomial nature of MNLFA limits its flexibility in modelling nonlinear moderation even when the functional form is known in some circumstances. In psychological science, moderation effects often have natural upper and lower bounds. Common examples include patterns of acceleration and deceleration observed in pubertal development (Marceau et al., 2011) and learning processes (Hipkins & Cowie, 2016). Despite the ability of the polynomial to approximate many nonlinear functions by increasing the polynomial order, higher‐order polynomial terms are typically unbounded. They thus cannot effectively represent natural bounds, especially for the points near the bounds (McNeish et al., 2023). Such terms can lead to infinitely increasing outputs with larger input values, resulting in unrealistic and impractical predictions in real‐world contexts. Typically, these natural bounds scenarios are characterised by sigmoid curves resembling elongated S‐shapes, which cannot be accurately captured by models with higher‐order polynomial terms. In addition, increasing the order of polynomial terms leads to difficulties in interpretation for applied researchers, complicating the practical application of the results (McNeish et al., 2023). Consequently, reliance on MNLFA risks biased conclusions for a sigmoid moderation effect with natural bounds, even if the functional form is known.

## LOCAL STRUCTURAL EQUATION MODELLING

To address the methodological challenges outlined above, Hildebrandt et al. (2009) proposed a nonparametric approach to modelling parameter changes in SEM under nonlinear moderation, termed LSEM (Hildebrandt et al., 2009, 2016). It should be noted that this method has also been abbreviated to LOSEM (Briley et al., 2015). Whereas some methodological descriptions with applications for LSEM exist, they are rather technical and not simply accessible for applied scientists. Thus, this tutorial provides a non‐technical explanation of the LSEM.

LSEM overcomes the limitations imposed by the categorisation of moderators, as seen in MG‐SEM. It operates on the principle of borrowing data from neighbouring values of the moderator to enrich the information at every focal point of a moderator of interest (Rey & Franklin, 2022), thereby allowing the modelling of SEM at each focal moderator point with adequate statistical power even if sample size at focal points is small. Moreover, this approach facilitates a detailed comparison of SEM parameters across values to describe the influence of a moderator. Beyond the function of MNLFA, LSEM does not assume any functional form of the moderation effect, making it a truly nonparametric method.

**Figure 1:**
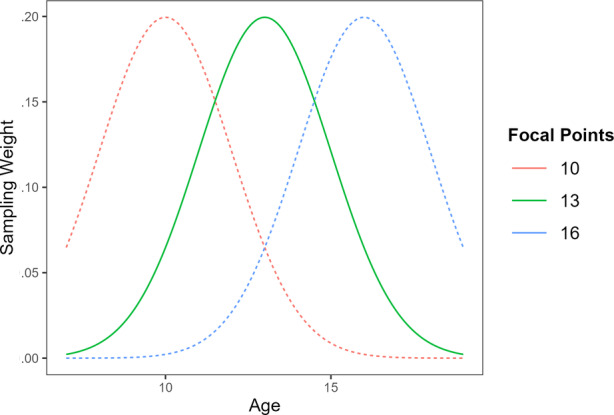
Plot of sampling weights with Gaussian Kernel (*h* = 2). *Note*: Gaussian kernel weighting functions for three selected focal age points used in LSEM. Only the weighting curves for focal ages 10, 13 and 16 are shown for clarity.

As described above, a central aspect of LSEM is the appropriate borrowing of data from neighbouring values of a moderator. The naive idea is to take the unweighted data directly from the *n*th nearest neighbours of the focal point of interest and use these to model the SEM. For example, to model SEM at age 15, data from ages 14 and 16 could be borrowed. However, this raises concerns about potential bias in SEM parameter estimation, as parameters at neighbouring values may not accurately reflect those at the focal point of interest. In this example, the information at ages 14 and 16 should not be the same as that at age 15. Nevertheless, information close to the focal point of a moderator should be more similar than distant information. In this example, the information at age 14 should be more similar to that at age 15 compared to that at age 5. Therefore, LSEM uses a weighted approach in which data closer to the target moderator values are given greater weight than those further away. Consequently, a weighted sample is created for each moderator focal point of interest, and the SEM model is then applied to each of these weighted samples. Typically, this weighting is implemented using a Gaussian kernel function that assigns the maximum weight of 1 at the focal point and progressively smaller weights to points increasingly distant from that focal point (Muller, 1987) (see Figure 1). The Gaussian kernel function's property of always yielding values greater than zero ensures that all data, regardless of distance from the focal point, contribute to the model at each value. However, distant data have a minimal practical impact on parameter estimation due to their lower weights (Olaru et al., 2023). Throughout the process, the researcher does not need to describe any possible parametric functional form of the moderation; therefore, this approach allows for a nuanced and localised nonparametric analysis of the nonlinear moderation effect within the SEM framework.

**Figure 2:**
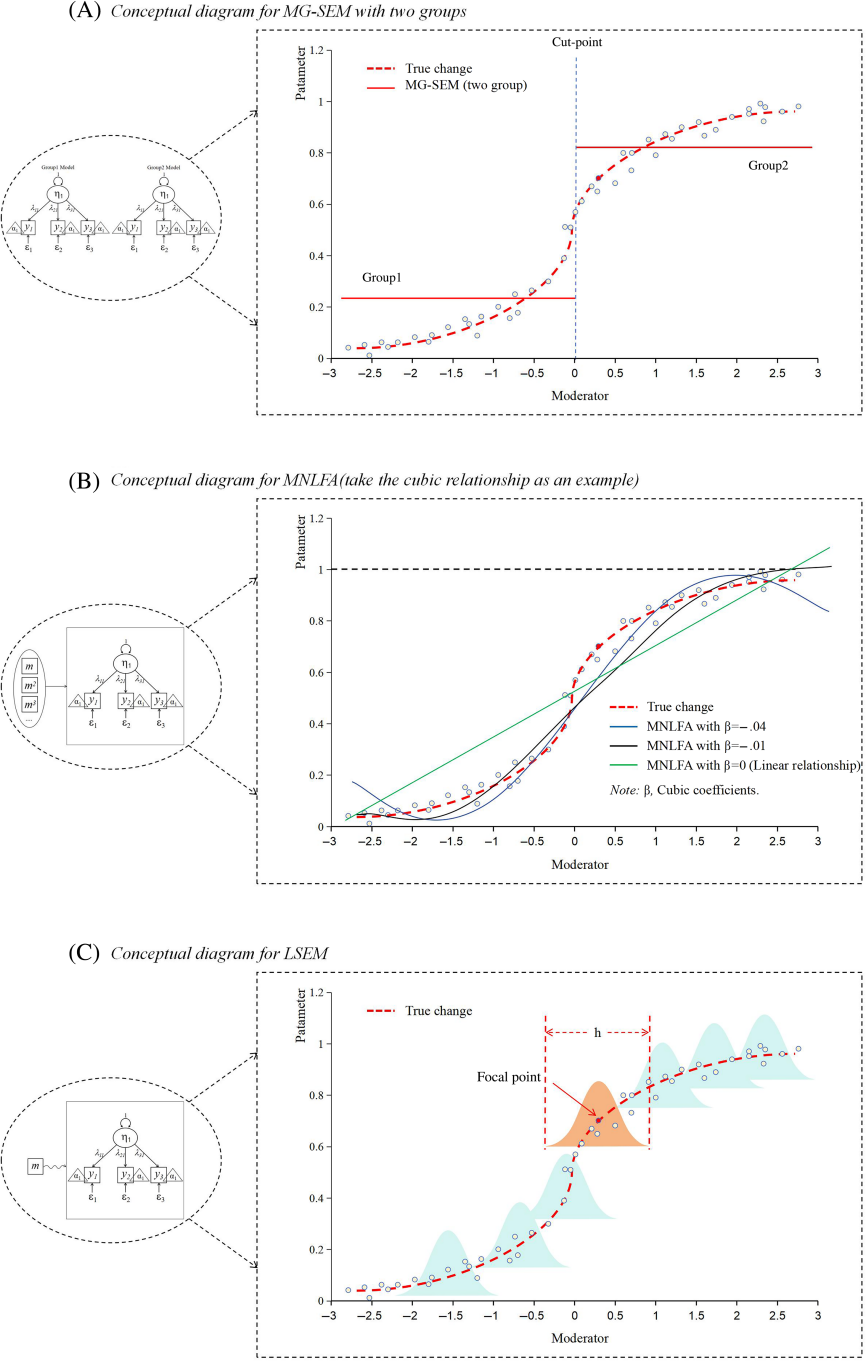
Conceptual diagram of *MG‐SEM*, *MNLFA,* and *LSEM*. *Note*: (a–c) illustrate three approaches figuratively. The actual pattern of the moderation effect was artificially created to follow an S‐shape and drawn as the red line. The dots in these plots represent each data point. The left part of this figure shows the conceptual example of SEM. η = latent factor; λ_*i*_ = factor loadings; *y*_*i*_ = manifest indicators; α_*i*_ = intercepts; ε_*i*_ = measurement error variances. (a) MG‐SEM results in a two‐group categorisation of the continuous moderator. (b) MNLFA modelled with pre‐defined functional form (here cubic) of the moderation effect. (c) LSEM operates on the principle of borrowing data (weighting using a Gaussian kernel with h as the smoothing parameter) from neighbouring values of the moderator.

To facilitate understanding of the three frameworks discussed previously (MG‐SEM, MNLFA, and LSEM) and their differences, we drew three conceptual diagrams (see Figure 2) that encapsulate the key characteristics of each framework. This visual representation serves as an intuitive guide, allowing applied scientists to grasp the essential aspects of each framework without reading the technical details in depth.

## EMPIRICAL ILLUSTRATION

After introducing the general idea of LSEM (for more technical details, see Hildebrandt et al., 2016; Robitzsch, 2023), this section demonstrates the implementation of LSEM through a developmental psychology example. The dataset in our example examines the relationship between parental depression and adolescent internalising problems. The dataset includes data from 19,564 parent‐adolescent pairs. Parenting depression was assessed through nine items, while adolescents' internalising problems were measured using five items. The moderator variable, age, was scored at 10 different age points, corresponding to ages 10–19, allowing for the analysis of 10 cross‐sectional age groups. This dataset is presented in the OSF repository (osf.io/ahe7s/).

LSEM has been implemented in the R statistical software using the sirt package. In the R statistical software, the lavaan package (Rosseel, 2012) is a comprehensive and widely used SEM package that accommodates a variety of models and estimation methods. The LSEM estimation functions as a wrapper for the SEM package lavaan, which means that the syntax of the LSEM model specification matches that of lavaan.

## FIRST APPLICATION SCENARIO: EXPLORATION WITHOUT PRIOR KNOWLEDGE

Previous research has consistently found a relationship between intense negative parental emotions, especially depression, and adolescent internalising problems (Fjermestad et al., 2017; Wilkinson et al., 2013). However, because adolescence spans a broad age range of approximately 10 to 20 years, it remains uncertain whether the relationship between parental depression and adolescent internalising problems is consistent across this age range. Specifically, the nature of this relationship's change—linear or nonlinear—with age remains unknown. Therefore, our primary goal is to exploratively examine the potential moderating effect of adolescent age on the relationship between parental depression and adolescent internalising problems without assuming a specific functional form of the moderation effect.

We began our analysis with a simple SEM model specification, modelling each construct as a latent variable and each item as a manifest indicator. For parenting depression, the model has nine intercepts, nine factor loadings, nine measurement error variances of the manifest indicators, and the variance and mean of the latent variable. Similarly, for adolescents' internalising problems, the model includes five intercepts, five factor loadings, five measurement error variances, and the corresponding variance and mean of the latent variable. In this tutorial, we applied an effect coding identification method to scale latent variables (Little et al., 2006). Different from the usually used unit loading identification (i.e., constraining the loading of one manifest indicator to one, intercept to zero) and unit variance identification (i.e., constraining the latent variance to one, latent mean to zero), effect coding identification avoids the problem of selecting an arbitrary and potentially noninvariant marker indicator. In addition, it does not require invariance of latent variance and mean. As a result, differences across the full range of moderators can be interpreted directly in manifest indicators, enhancing the clarity of the results. Afterward, an additional parameter, the structural correlation path, was estimated to examine the relationship between parental depression and adolescent internalising problems.

In SEM, the concept of measurement invariance, which ensures that latent variable scores maintain consistent meaning and metrics across individuals (Bauer et al., 2020), is critical. Measurement invariance in SEM is typically defined at four levels (Gana & Broc, 2019): equal model structures (configural invariance), equal factor loadings (metric invariance), equal intercepts (scalar invariance), and equal measurement error variances of manifest indicators (strict invariance). Previous literature emphasises the need to account for measurement non‐invariance when examining moderation effects (Guenole & Brown, 2014; Maassen et al., 2023), although there is debate in the field (Funder & Gardiner, 2024; Robitzsch & Lüdtke, 2023). For example, a simulation study by Hsiao and Lai (2018) shows that measurement non‐invariance significantly affects the moderation effect estimation in MG‐SEM. Measurement invariance is also relevant in nonlinear moderation contexts such as MNLFA, where non‐invariance in the measurement model could confound structural‐level interpretations (Bauer, 2017). Therefore, while measurement invariance is not the primary focus of this tutorial, some researchers are recommended to establish scalar or partial scalar invariance in the measurement model, at least, before conducting moderation analyses. This is because scalar invariance is the minimum level required for comparing latent means and variances across participants (van de Schoot et al., 2012).

Testing for measurement invariance integrates seamlessly into the LSEM framework because measurement invariance can be modelled as a specific type of moderation that affects only measurement model parameters (e.g., intercepts, factor loadings, measurement error variance) across moderator values (Hildebrandt et al., 2016). For simplicity, in this tutorial, we assume scalar invariance of both latent variables across the different values of adolescent age, implying no age‐related moderation effect on the intercepts and factor loadings of all manifest indicators. For a comprehensive guideline of measurement invariance testing using LSEM, we refer the reader to Robitzsch (2023). The code for testing measurement invariance in our example is available in the OSF repository (osf.io/ahe7s/) as supplemental materials.

### Implementation

To run the LSEM analysis, we use the lsem.estimate() function in sirt (Robitzsch, 2024). There are several key steps in preparing the arguments for this function. First, an SEM model is specified using lavaan syntax. In this scenario, we specified a lavaan syntax (see code in the supplemental material) and put it as the lavmodel argument in this function. The second step is to define the moderator. In our case study, we moderated the SEM across adolescents aged 10 to 19 in one‐year increments, putting seq(10, 19, 1) as the moderator.grid argument. Researchers can adjust the granularity of this step as needed, for example, each increment at two age points. Afterward, setting the bandwidth parameter as the input in the h argument, which acts as a smoothing parameter in the Gaussian kernel weighting function, is important. A larger bandwidth means that the neighbours of the focal point are assigned a higher weight, resulting in smoother estimates. In the limiting case where the bandwidth approaches infinity, resulting estimates converge to those of a conventional single‐group SEM that does not account for moderation effects. Therefore, choosing the optimal bandwidth requires balancing a model that is too smooth and lacks nuanced detail against a model that is too detailed but potentially overfitted (Robitzsch, 2023). Following the recommendations of previous studies (Hildebrandt et al., 2016; Robitzsch, 2023), we chose a bandwidth of h = 2, shown by simulation to be effective in the SEM context. As a wrapper for lavaan, standard lavaan arguments can be used directly. For example, we used std.lv = TRUE to apply the unit variance identification constraint, used standardised = TRUE to export standardised coefficient results, and added meanstructure = TRUE to include intercepts for manifest indicators, and the latent variable mean. As described before, we assumed scalar invariance for both latent variables, using the par_invariant argument to ensure equal factor loadings and intercepts across ages (see code in the supplemental material for more details). When the model includes invariant parameters, LSEM automatically employs a pseudo‐multiple group model within a joint estimation approach, using a pseudo‐likelihood function across moderator values for simultaneous parameter estimation. This provides a global model fit (RMSEA, CFI and SRMR) across all moderator values rather than separate model fit indices for each value. This joint estimation approach is similar to that used in MG‐SEM and allows for comparing constrained and unconstrained models by comparing the model fit. Joint estimation is generally preferable unless specific needs arise. The code for running LSEM in the tutorial is detailed below, and more information on the function's arguments is available in the sirt package manual or Robitzsch (2023).




lsem <‐ lsem.estimate(data = data, lavmodel = SEM_model, moderator = "cage", moderator.grid = seq(10, 19, 1), h = 2, standardized = TRUE, meanstructure = TRUE, par_invariant = MI_constraints)




To explore the moderation effect on a particular parameter, the significance test can be used to evaluate the equivalence of parameters across moderator values. More specifically, the variances of particular parameters at different moderator values are considered a sample of parameters, where the significance test of parameter equivalence across moderator values can be viewed as a one‐sample t‐test with the null hypothesis that the population of parameter variances is zero. Calculating the empirical *t*‐value requires calculating the standard deviation of this parameter variance sample; however, traditional methods might be inappropriate here due to the overlapping weighted samples used in LSEM. Therefore, nonparametric bootstrapping is proposed as an alternative method to estimate the standard deviation (Robitzsch, 2023) unbiasedly. The function lsem.bootstrap() performs this, where the R argument specifies the expected number of bootstrap samples. While the default value is 100 to obtain standard error for normally distributed parameters, more bootstrap samples are recommended for more robust results (Mak, 2004). It should be noted that bootstrapping is computationally very intensive; therefore, parallel computing can be used to speed up the calculation. The number of cores for parallel computing depends on the analysis computer and can be specified by the n.core argument. In our example, we used four cores, but more can be used to reduce computation time. The code used to implement the bootstrapping is as follows.




b_lsem <‐ lsem.bootstrap(object = lsem, R = 100, n.core = 4)
plot(b_lsem, parindex = 62)




**Figure 3:**
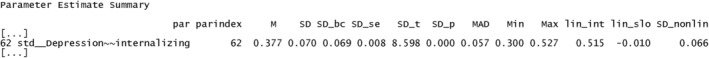
Part of the output of lsem.bootstrap() for the illustrative dataset. *Note: par* = parameters, parindex = indices for parameters; *M* = the estimated mean; *SD* = the sample estimated standard deviation (biased due to weighted samples overlapping); *SD_bc* = the estimated standard deviation using bootstrapping; *SD_se* = the estimated standard error; *SD_t* = the estimated *t*‐value; *SD_p* = the *p*‐value of estimated variance of parameter (calculated by one‐sample *t*‐test); MAD = mean absolute deviation of parameter; Min = the minimum value of parameter across the moderator; Max = the maximum value of parameter across the moderator. Other values mentioned are beyond the scope of this analysis.

**Figure 4:**
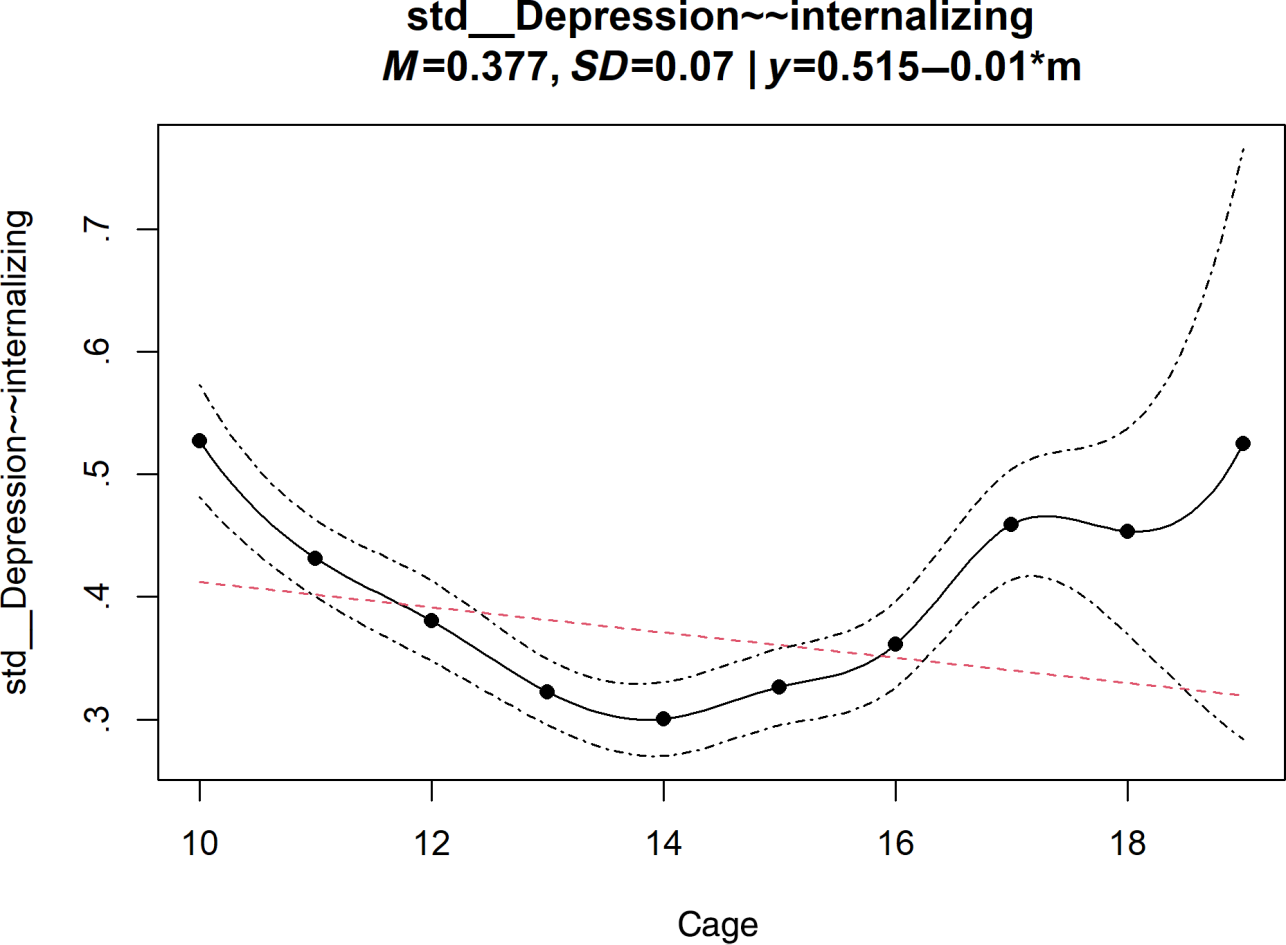
Parameter curves for the standardised latent correlation from LSEM analysis for the illustrative dataset.

The results (Figure 3) can be reviewed using the summary() function on the output of lsem.bootstrap(). For example, From line 62 in our example, we observed significant variation in the standardised coefficient of the latent correlation between parental depression and adolescent internalising problems (std__Depression~~internalizing) across adolescent ages (SD_bc = 0.069, *t* = 8.598, *p* < .001). This indicates a significant change in the correlation across adolescent ages. By examining the output graphs of the plot()function on the output of lsem.bootstrap(), we can explore the patterns of the moderation effect. The parindex argument specifies the parameter to be plotted; in our case, the parameter number is 62, representing the line number in the output from the summary()function. By visually inspecting this plot (Figure 4), we could see an obvious nonlinear change in the correlation between parental depression and adolescents' internalising problems across ages. The confidence intervals in this plot are crucial for interpreting parameter significant differences; if point estimates at one moderator value fall outside the confidence intervals of another, significant differences between these two points can be inferred. For example, our analysis revealed a significantly lower correlation at age 14 than at ages 10 and 18.

## SECOND APPLICATION SCENARIO: CONFIRMATORY TESTING A HYPOTHESISED FORM OF MODERATION

We aimed to clarify that LSEM goes beyond a pure exploratory analysis, although its nonparametric nature makes it well suited for examining moderation effects without prior knowledge. LSEM can also be used for hypothesis‐driven investigations, like MNLFA, by pre‐specifying and testing a parametric nonlinear moderation effect. In other words, we could hypothesise a parameter of interest in SEM as a specific parametric function (linear or quadratic) of a moderator and test whether this parametric function is sufficient to describe the moderation effect. Again, we used the same data as in the scenario for simplicity.

Consider research that proposed a potential U‐shaped change between parental depression and adolescent internalising problems with age development. In other words, this research hypothesised an initial high correlation between the two latent variables in early adolescence, following a decrease in middle adolescence and a subsequent increase in late adolescence. To test this hypothesis, we could use LSEM to evaluate the linear and quadratic coefficients of this proposed moderation change. In line with our first scenario, the first step was to run an LSEM model and assess the significance of the variation in the parameter through bootstrapping, which is fundamental to our analysis. Afterward, we modelled the parameter sample and regressed it on the moderator. Rather than using a one‐sample *t*‐test to test for parameter equivalence across values of a moderator, we specified a particular parametric function based on the theoretical hypothesis.

### Implementation

Here, we used the lsem.test() function. The particular parametric function was specified to test the significance of the moderator's linear and quadratic regression coefficients on this latent correlation. However, unlike means, intercepts and loadings, correlations cannot be modelled directly by a parametric moderation function without transformation because their absolute values cannot exceed one. To address this, Fisher's z‐transformation is applied, as Bauer (2017) recommended, to constrain the correlations within the bounds of −1 and 1. The syntax for implementing this in the lsem.test() function would be: “std__Depression~~internalizing” = 0.5 × log((1 + y)/(1 − y)) ~ m + I(m^2^). Here, the left side of the tilde represents Fisher's *z*‐transformation of the correlation, and the right side is consistent with standard polynomial regression in R and can be explained similarly to the MNLFA. Then, the specified function syntax is entered in the models argument. In addition, the outputs of the lsem.estimate() and lsem.bootstrap() functions are included in the mod and bmod arguments. The code used is as follows:




Test <‐ list("std__Depression~~internalizing" = 0.5 * log((1 + y) / (1 ‐ y)) ~ m + I(mˆ2))
tmod <‐ lsem.test(mod = lsem, bmod = b_lsem, models = Test)




**Figure 5:**
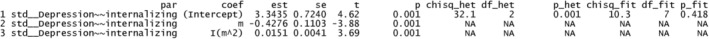
Part of the output of lsem.test() for the illustrative dataset. *Note: par* = parameters; *coef* = coefficients for the pre‐specifying moderation effects; *est* = the estimated value of coefficients; *se* = the sample estimated standard error of coefficients; *t* = the estimated *t*‐value of coefficients; *p* = the *p*‐value of coefficients (calculated by *t*‐test); *chisq_het* = the global test for parameter heterogeneity; *df_het* = the degree of freedom of the global test for parameter heterogeneity (same to the number of coefficients); *p_het* = the *p*‐value of the global test for parameter heterogeneity; *chisq_fit* = the test of a sufficient fit of a parameter curve; *df_fit* = the degree of freedom of the test of a sufficient fit of a parameter curve; *p_fit* = the *p*‐value of the test of a sufficient fit of a parameter curve.

The results of lsem.test() can be accessed with print(tmod$test_models). This result can be interpreted in the same way as a regression analysis. The test for parameter heterogeneity (chisq_het) evaluates the overall significance of all coefficients compared to the only intercept model, while individual *t*‐tests examine the significance of each coefficient separately. In our example (Figure 5), the whole model was statistically significant compared to only the intercept model (*p* < .001). Afterward, we could read from lines two and three that both the linear and quadratic regression coefficients were statistically significant (b_m = −0.428, *t* = −3.88, *p* < .001; b_I(m^2^) = 0.015, *t* = 3.69, *p* < .001), with the quadratic coefficient being positive, indicating a U‐shaped trend of changing. Most importantly, the test of sufficient fit (chisq_fit) is not significant (*p* = .418), indicating no significant difference between the non‐parametric nonlinear local fit and the hypothesised parametric nonlinear function in describing this moderation effect. Combined with the plot function described earlier, this result suggested that the hypothesised (quadratic) nonlinear moderation effect was supported. This example demonstrates the utility of LSEM not only as an exploratory approach but also for confirmatory analysis, similar to the application in MNLFA.

## OTHER ISSUES IN MODELLING: ALTERNATIVE METHOD FOR BOOTSTRAPPING

Bootstrapping need be used to assess the significance of parameter change across different moderator values, as described previously. However, alternative approaches, such as permutation testing, have been used in many previous studies, such as Olaru et al. (2019) and Basarkod et al. (2023). In permutation testing, parameters sampled across the values of a moderator are compared to a distribution that would be expected from sampling error alone (Huo et al., 2014). This is accomplished by generating numerous resampled copies of the dataset and randomly shuffling the moderator values across individuals (Hülür et al., 2011; Jorgensen et al., 2018). This process effectively nullifies any systematic moderation effects in the data, resulting in a distribution of parameter estimates independent of the moderator. The original LSEM parameter estimates are then compared to this null distribution, providing *p*‐values for each model parameter along the moderator, allowing researchers to identify which parameters change significantly across the values of a moderator. More technical information on the permutation test can be found in the paper by Hildebrandt et al. (2016).

In R, we used lsem.permutationTest() function for this purpose. This function takes the output of the lsem.estimate() function as input. The B argument sets the number of permutation samples with a default of 1000. Like bootstrapping, permutation testing is computationally intensive, so parallel computing is recommended to speed up the process. The code used is as follows:




permutation <‐ lsem.permutationTest(lsem.object = lsem, B = 1000, n.core=4)




**Figure 6:**
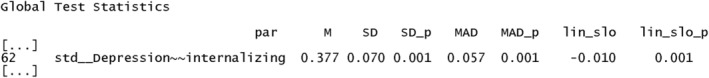
Part of the output of lsem.permutationTest() for the illustrative dataset. *Note: par* = parameters; *M* = the estimated mean; *SD* = the estimated standard deviation using permutation; *SD_p* = the *p*‐value of estimated variance of parameter (calculated by permutation test); MAD = mean absolute deviation of parameter; *MAD_p* = the *p*‐value of estimated mean absolute deviation (calculated by permutation test). Other values mentioned are beyond the scope of this analysis.

The results (see Figure 6) can be examined using the summary() function. As with bootstrapping, permutation testing again indicated significant variation in the latent correlation between parental depression and adolescent internalising problems across adolescent ages (*p* < .001).

Unlike bootstrapping, permutation testing does not depend on *t*‐statistics, which assumes a normal distribution of parameter samples across moderator values (Robitzsch, 2023). This means permutation testing can produce unbiased results regardless of the parameter distribution across moderator values. Permutation testing assumes that all parameters remain invariant, unlike the bootstrap approach. Consequently, it is more computationally intensive than bootstrapping because it simultaneously examines the moderation effects of all parameters. Moreover, permutation testing is less suitable for evaluating moderation effects based on complex parametric functions for confirmatory testing of a hypothesised moderation effect (Olaru et al., 2023), as demonstrated in our second scenario. Therefore, we recommend bootstrapping in most cases, except when the normality of the parameter samples is in question.

### Missing values

Missing data is a common problem in research (Olinsky et al., 2003) and arises from different mechanisms: missing completely at random (MCAR), missing at random (MAR) and nonignorable (named as not missing at random, NMAR), each of which requires different handling strategies (Dai, 2021). In SEM, pairwise deletion is commonly used for the data with MCAR (Allison, 2003), despite potential bias. Full information maximum likelihood (FIML) is usually recommended for MAR data (Enders & Bandalos, 2001), as well as for MCAR data to improve efficiency. Even when the missing mechanisms are nonignorable, FIML estimates tend to be the least biased (Schreiber, 2008).

In LSEM, missing can occur in the moderator or any of the manifest indicators of the SEM. Typically, LSEM handles missing manifest indicator data by pairwise deletion. However, this approach can lead to biased results if the missing are not MCAR. For MAR, FIML can be seamlessly integrated into LSEM using the lsem.estimate() function in R by simply adding the missing = "ML" argument. While FIML is alredy used in LSEM research, such as Hartung et al. (2021), it has limitations that can lead to biased LSEM estimation. Applying FIML simultaneously to the mean structure and covariance matrices may confound the estimation of covariance matrices due to trends in the mean structure (Robitzsch, 2023). An alternative is multiple imputation, which has been shown to be as effective as FIML in SEM (Collins et al., 2001). However, integrating multiple imputation results using Rubin's rules remains complex in LSEM (Basarkod et al., 2023). Therefore, our recommendation is that the choice between FIML and multiple imputation depends on the research focus. If mean structure estimation is not a priority, FIML is a suitable option with the meanstructure = FALSE argument. This setting ensures that FIML is applied solely to covariance estimation with detrending the mean structure. In contrast, if the mean structure is important, multiple imputations should be considered, with the caveat that the results of each imputed dataset would be presented individually, similar to a manual “cross‐validation” process. This “combined” strategy maximises parameter estimation accuracy in LSEM analysis and accommodates the complexities inherent in LSEM analysis with missing data. For missing in moderators, multiple imputation remains the only viable solution because FIML cannot handle missing data outside of the SEM model. Nevertheless, further research is needed to understand the implications of missing moderator data in LSEM.

## OTHER APPLICATION SCENARIOS

The versatility of LSEM is not only limited to the scenarios presented earlier but also to other more complex research scenarios. This section aims to broaden the scope of our tutorial for applied scientists by highlighting its potential in different scenarios. We refer to studies that have extended the LSEM framework to accommodate complex research scenarios. These references are academic citations and resources for researchers facing similar challenges. We encourage readers to explore these works in depth to grasp how LSEM's versatility can be adapted to different research needs.

### Multiple moderators or latent moderators

LSEM is not limited to using age as the sole continuous moderator, as in our example. Similar to MNLFA, LSEM can also include multiple different types of moderators to examine complex patterns of moderation effects simultaneously. For example, to include a categorical moderator and a continuous moderator and their interaction simultaneously, researchers can combine LSEM with MG‐SEM as a hybrid approach. Specifically, an LSEM model can be built for each focal point of the continuous moderator, and within each point, MG‐SEM could be used to account for the categorical moderator. When dealing with multiple continuous moderators, the Gaussian kernel function could be replaced by a multivariate Gaussian kernel function to compute weights, as shown in Hartung et al. (2018), which can capture potential interaction effects between multiple moderators. The inherent nonparametric nature of LSEM provides greater flexibility in modelling these interactions compared to MNLFA, as the interactions do not need to be constrained to a predefined parametric form.

Another useful extension in LSEM is incorporating a latent moderator, such as ability, personality traits or socio‐economic status, which is typically measured using multiple manifest indicators. In LSEM, moderators must be a manifest variable. Thus, a two‐step approach is recommended for including the latent moderator in the analysis. First, researchers can use item response theory (IRT), factor models (for reflective measurement model) or principal component analysis (for formative measurement model) to estimate latent factor or component scores. This manifest score is then incorporated into the LSEM as a moderator. An illustrative example can be found in the study by Liu et al. (2022).

### Advanced SEM model and categorical indicators

LSEM is also not limited to a simple SEM model with only two latent factors, as in our example. It can also be applied to more sophisticated SEM models. The lsem.estimate() function in R, which acts as a wrapper for the widely used lavaan package, facilitates the integration of LSEM with various advanced SEM models using lavaan syntax. Given the broad range of models lavaan supports, we highlight only a few potential applications. For cross‐sectional data, LSEM has been used with high‐order or bifactor models (Schroeders & Jansen, 2022). In longitudinal data analysis, it has been used with latent growth curve models (Brandt et al., 2023; Olaru et al., 2023) and latent change score models (Olaru & Allemand, 2022). In addition, to address nested data structures, Hartung et al. (2020) incorporated the sandwich estimator into LSEM to account for the non‐independence of observations within clusters.

While our example treats the manifest indicators as continuous variables, LSEM can also model categorical or ordinal manifest indicators. One approach is to use the weighted least squares mean and variance adjusted (WLSMV) or maximum likelihood with robust standard errors (MLR) estimators available in lavaan for handling categorical data (Li, 2016), as shown in Bolsinova and Molenaar (2019) and Panayiotou et al. (2023). Another possibility is to combine IRT with LSEM. Although this has not yet been modelled in previous studies, it is feasible given that the principle of local estimation based on weights can be extended to any model compatible with sampling weights, such as the mirt package for multidimensional IRT (Chalmers, 2012).

### Psychometric network modelling

In recent years, psychometric network modelling has emerged as an innovative approach that differs from conventional psychometric approaches such as SEM and IRT (Hevey, 2018). This novel approach differs fundamentally in its framework. Instead of viewing constructs as latent factors causing manifest indicators, psychometric network modelling treats indicators as systems of interrelated components without assuming underlying latent factors (Lange et al., 2020). By emphasising the direct connections between manifest indicators, psychometric network modelling provides a distinctive lens through which the structure and dynamics of constructs can be explored, potentially leading to novel insights and interventions (Borsboom & Cramer, 2013).

Similar to SEM, the principles of LSEM can be adapted to psychometric network modelling, leading to the development of local psychometric network analysis (Hartung et al., 2020). However, a difference between psychometric network modelling and SEM is the regularisation techniques (Robinaugh et al., 2020) to select the most relevant paths, a feature prevalent in but not typically present in traditional SEM (even though regularised SEM exists). The implications of incorporating a weighting function in this context, particularly with respect to statistical inference of parameters in a psychometric network model with regularisation techniques, need future research. Despite this challenge, as an exploratory tool consistent with the spirit of psychometric network analysis (Borsboom, 2022), the integration of LSEM into network modelling has shown promise, as evidenced by studies such as Madole et al. (2019) and Walters and Simons (2022).

## LIMITATION OF LSEM

While LSEM has many advantages, its limitations should be acknowledged. A key limitation, as highlighted throughout this tutorial, is the computational intensity of LSEM, especially when using bootstrapping for statistical inference. This aspect can significantly increase the time and resources required for analysis. Recently, a mixture modelling‐based approach to LSEM has been developed that does not require bootstrapping (Molenaar, 2021), potentially lessening the computational burden. However, as this is a relatively new method, more research is needed to compare this approach with previous LSEM methods regarding efficiency. In addition, as a nonparametric method, LSEM generally requires a larger sample size to achieve the same level of statistical power as parametric methods such as MNLFA under comparable conditions. This means that in scenarios where the parametric form of the moderator's effect on the parameters is known, MNLFA may provide greater statistical power in testing the significance of parameter regression coefficients than the nonparametric LSEM approach (Robitzsch, 2023). Despite these challenges, using LSEM is still highly recommended, especially when considering the risks associated with the misspecification of the parametric form in other methods. LSEM's flexibility and adaptability to a wide range of research scenarios make it a valuable tool in the applied scientists' toolkit, albeit with an understanding of its computational demands and sample size requirements. This balanced view encourages scientists to consider LSEM thoughtfully in their research practice, leveraging its strengths while being mindful of its limitations.

## CONCLUSIONS

This tutorial introduces LSEM as a flexible and versatile framework for investigating nonlinear moderation effects in SEM, which is often overlooked in traditional linear frameworks. As a nonparametric method, LSEM offers a methodological advance over previous SEM moderation approaches by eliminating the need to categorise moderator variables and avoiding reliance on predefined parametric function forms of moderation effects. The sirt package in R provides user‐friendly functions that enable applied researchers to investigate and test for nonlinear moderation in SEM easily. Through two application scenarios, we demonstrate that LSEM is adept at exploring nonlinear moderation without prior knowledge and confirmatory testing a hypothesised form of parametric moderation. We advocate LSEM as a valuable tool for applied psychological scientists and encourage its implementation in diverse research practices.

## COMPLIANCE WITH ETHICAL STANDARDS

All procedures performed in studies involving human participants were in accordance with the ethical standards of the institutional research committees. All the participants provided the informed consent.

## Data Availability

The data and analysis code are available in the OSF repository (osf.io/ahe7s/).
